# Record linkage studies of drug-related deaths among former adult prisoners who have been released to the community: a scoping review.

**DOI:** 10.23889/ijpds.v7i3.2044

**Published:** 2022-08-25

**Authors:** Janine Cooper, Ifeoma Onyeka, Dermot O'Reilly, Richard Kirk, Michael Donnelly

**Affiliations:** 1 Queen's University Belfast; 2 South Eastern Health and Social Care Trust

## Objectives

Substance use disorders are common in prisoners before they are admitted to prison. Epidemiological studies have demonstrated an increased risk of death following release from prison. The aim of this scoping review is to identify, map and summarise evidence from record linkage studies about drug-related deaths among former adult prisoners.

## Approach

We followed the framework for conducting scoping reviews by Arksey and O’Malley, and guidance by the Joanna Briggs Institute. MEDLINE, EMBASE, PsychINFO and Web of Science were searched for studies from January 2011 to September 2021 using keywords and index headings relating to ‘mortality’, ‘drugs’ and ‘ex-prisoner’. Two authors independently screened titles and abstracts for eligibility using pre-defined inclusion criteria. Full publication screening for inclusion was performed independently by two authors. Two authors tested the charting form and are independently extracting information from the eligible publications using this form. There were no geographical restrictions but non-English language papers were excluded.

## Results

Following the removal of duplicates, 3680 publications were screened using title and abstract. Full text publications were subsequently screened for eligibility. A total of 48 publications have been included in this review. Publications may be added from a search of reference lists. Two authors are performing data extraction and data will be presented and analysed in relation to answering the following scoping review questions:

1. What is the scope of the literature on record linkage studies of drug-related deaths among former adult prisoners who have been released to the community?
2. How is research conducted on this topic?
3. What methodologies are used?
4. What are the findings in relation to mortality?
5. Where are the knowledge gaps on this topic?

## Conclusion

This review will help identify and profile at risk former prisoners, contribute to potential interventions, and inform future research and policy. Where possible, evidence will be reported about the time period to death after prison release and the specific drugs related to mortality.

